# Introducing a multimodal perspective to emotional variables in second language acquisition education: Systemic functional multimodal discourse analysis

**DOI:** 10.3389/fpsyg.2022.1016441

**Published:** 2022-10-17

**Authors:** Zhengyuan Liu

**Affiliations:** Foreign Languages School, Huanghuai University, Zhumadian, China

**Keywords:** systemic functional multimodal discourse analysis, semiotic resources, complexity and dynamic systems theory, visual language, affective variables, second language acquisition

## Abstract

A systemic functional theory applied to multimodal discourse analysis (SF-MDA) is related to the theoretical and practical meaning extraction from the data showing how multiple semiotic resources are used in different modes of discourse ranging from textual, printed, and electronic texts to visual language and those existing alive as embedded in reality. In the present paper, we provide an introduction to the SF-MDA approach and then justify how it can benefit the second language acquisition (SLA) domain and more specifically the exploration of L2 affective variables. We will show how the SF-MDA approach fits in with the complexity and dynamic systems theory (CDST), and is capable of revealing the nuances of the developmental nature of the affective variables involved in language learning. The exemplary works of research in the SLA domain using the SF-MDA approach are reviewed here as well as those exploring L2 affective variables. The further benefits of this approach will be emphasized at the end along with the implications it can have for the CDST-led line of research in applied linguistics.

## Introduction

The new technological advancements, in particular, in the area of computers, education, and related stakeholders have been seeking to integrate visual or aural elements into SL/FL programs This has made it possible for learners to gain easier access to multisemiotic resources than before (Wang and Hemchua, 2022). Undoubtedly, regardless of any subject at school, an attempt has been made by the material developers to incorporate multimodal resources during the last 30 years (Unsworth, 2001). Along the same line, the semiotic approach attaches great importance to the functional role of images as potential vehicles of cultural elements accompanied by texts and pedagogic tasks; that is, these pictures are not merely decorative elements (Derakhshan, 2021). The semiotic perspective focuses on the potential resources of textbooks and can be used as a tool for figuring out how much and how the books can represent the cultural elements. Based on this approach, culture is an outcome of a dynamic process created in educational settings. This process unfolds as a result of interactions between students and instructional materials, highlighting the processes learners undergo in this context (Kiss and Weninger, 2017).

As pointed out by Kramsch (2013), the semiotic perspective is based on the postmodernist view and analyses the inclusion of cultural dimensions in learning materials in a dynamic manner. It also draws on interactive, multimodal considerations. Indeed, this approach views culture as a discourse, or what is known as a social semiotic construction. Using a flexible procedure, this approach probes the development of particular cultural meanings by integrating the texts, tasks, and pictures, using semiosis for achieving curriculum goals, and highlighting the cultural elements. These resources make it easier for the learners to read and understand the cultural contents (Chen, 2010; Derakhshan, 2021). A systemic functional approach to multimodal discourse analysis (SF-MDA) entails employing practical and theoretical approaches to analyzing textual input, three-dimensional materials or other activity types marked by a combination of semiotic qualities (e.g., written and spoken language, mathematical symbolism, visual input, architecture, gesture, sculpture, or other physiological modes) for meaning-making (O’Halloran, 2008). The systemic functional theory is appropriate for the provision of theoretical foundations for multimodal discourse analysis (MDA) as, most importantly; it is a theory of social semiotics that sees meaning largely contingent on context (Halliday, 1978). Until now, the systemic functional theory has acted as an insightful theory of language (e.g., Martin, 1992; Halliday and Matthiessen, 1999; Martin and Rose, 2003; Halliday, 2004). Influenced by the language of displayed art and Kress by O’Toole (1994) and the reading images: the grammar of visual design by Van Leeuwen (1999), attention has been more increasingly attracted to SF theory applied for MDA (e.g., Lemke, 1998; Royce, 1998; Van Leeuwen, 1999, 2005; Iedema, 2001, 2003; Kress and Van Leeuwen, 2001; Unsworth, 2001; Martin, 2002; O’Halloran, 2004, 2005; Ventola et al., 2004; Martinec, 2005; Baldry and Thibault, 2006; Bateman, 2008). The foundation of SF-MDA further development and the subsequent issues are related to the systemic functional theory quality.

Thus, the multimodal aspects of emotions from an SFMA perspective can be realized in terms of experiential, multimodal, and textual functions. The main advantage of SF-MDA is the meta-functional principle established by Halliday (1978, 2004) considered the foundation of theorizing about the interaction of semiotic resources meaning-making (e.g., Van Leeuwen, 1999, 2005; Baldry and Thibault, 2006; Kress and van Leeuwen, 2006). That is, compared to other approaches to multimodal analysis such as MDA, SF-MDA is unique as it categorizes the multimodal aspects of classroom interactions into three main functions. One of the main advantages associated with SF theory has to do with the metafictional principle developed by Halliday (2004). This principle lays the groundwork for postulating the way in which the interaction of semiotic resources yields meaning. Here, these concepts need to be clarified: semiotic elements, modality, and multimodal resources (Kress and van Leeuwen, 2006). O’Halloran (2011) maintains that semiotic resources help the students to make meaning. Some of these resources are as follows: language, pictures, and music. Modality refers to the sensory channel used for communication (e.g., visual, auditory, tactile, and kinesthetic). There is a close interrelationship between these two concepts. For example, the L2 classroom should take advantage of a variety of semiotic resources, such as language and digitalized pictures, films, and the instructor’s gestures and body language (Levasseur and Sawyer, 2006). These semiotic elements improve the learners’ capabilities, including their visual, aural, and/or kinesthetic sensory stimulation. Therefore, multimodality has been used as a term with a broad sense involving several semiotics and modalities (Rowsell and Collier, 2017). According to the meta-functional rule, the semiotic assets both set the stage for ideational meaning-making (i.e., logical relations and experiential meaning) and pave the way for establishing social associations (i.e., inter-individual meaning). Such meta-functions are activated within the discourse structure, considered as a textual semiotic meta-function.

The meta-functional principle acts as a platform of exploring the uses of semiotic assets and analyzing how semiotic resources are implicated together in discourses of multiple modes to serve certain purposes (e.g., for meaning-making in school coursebooks, to encourage a customer to buy a certain property, to encourage people to act violently apparently for some other reason) (O’Halloran, 2008). What matters in SF-MDA is the meaning value of semiotic assets shared among different levels (i.e., phonology and lexicogrammar, graphology/typography, setting, and discourse semantics) and the theoretical investigation of the coordinated meaning of semiotic alternatives in some discourse marked by multimodality (O’Halloran, 2008). Multimodality is concerned with several approaches taken to represent the communication of things by resources other than language. Here, multiple communicational modes such as picture, body language, and movements, among others, or integration thereof are used (Jewitt, 2011). Indeed, linguistic and visual modes of semiosis are fundamentally different. For example, a linguistic resource, i.e., a text, is usually arranged syntactically as a chain that is sequentially processed, with meaning-making progressing progressively. SFL requires the analysis of the sequential parts (i.e., the lexical items, sets of words, clauses, clauses, and paragraphs), which constitute several stages in textual development (O’Halloran, 2008). SFL involves the individual’s ability to interpret texts with respect to the context where these texts are created and received. Indeed, SFL views context as a factor consisting of three language registers: field of discourse associated with the experiential meanings in question; tenor of discourse that attaches great importance to the formation of social relations and functions; and mode of discourse, which is concerned with how semiotic forms are arranged and presented. The latter is manifested in texts through the application of thematic elements. The integration of these modes contributes to the organization of the informational function of a text, making it a coherent whole (Alyousef, 2020). As one of its important advantages, SFL conceptualizes language as one of the multiple interrelated semiotic systems which makes a contribution to the development of intersemiotic theory. Other systems include mathematics, animation, poetry, mimicking, and music, among others. Such a framework should accommodate new forms of communication (Unsworth, 2006).

For example, the SF-MDA approach contributed to the investigation of the functional properties of language, optical input and arithmetic symbolism of discourse (O’Halloran, 2008). It also deals with theorizing how visual, linguistic, and interactional symbolic alternatives work together for reality construction in ways that go beyond the superficial uses of mere linguistic assets (O’Halloran, 2005, 2007). Moreover, SF-MDA tries to link multiple disciplines which are from conventionally different domains. The SF-MDA perspective can significantly contribute to the conceptualization and analysis of multi-modal semiotics (O’Halloran, 2008). As with Systemic Functional Theory, in the case of SF-MDA, a combination of resources, including system networks and meaning represent the meanings associated with semiotic resources. Here, the meanings also unfold metafunctionally (Lim, 2021). Since classroom interactions in language classes have a multimodal nature and emotions arise out of these multimodal interactions, the multimodality underpinning these emotions should be taken into consideration. In the second language acquisition (SLA) domain, SF-MDA can be effectively used to explore multimodal classroom learning experiences, as will be addressed below.

## Review of the literature

### Contribution of systemic functional-multimodal discourse analysis to second language acquisition domain

Derived from Martin and White’s (2005) appraisal theory, SF-MDA has its roots in Halliday’s (1985) systemic functional linguistics including textual, interpersonal, experiential, and meta-functional inputs. SF-MDA has recently been used in the applied linguistics field (Peng et al., 2017; Erfanian Mohammadi et al., 2019). Multimodal experiential meaning is categorized as contexts, the participants involved, and the process (Hood, 2011; Lim, 2011). By process, we mean the participants’ conditions (e.g., sitting, standing), the process of their behavior (e.g., whether they are showing enjoyment or boredom), their interaction with learning resources (e.g., tasks and activities), and their physical responses (e.g., facial expressions, nodding).

Multimodal interpersonal meaning is divided into engagement, attitude, and graduation. Attitudes can be considered to be either negative or positive. Engagement is context-dependent. Several body expressions such as the thumbs-up show positive attitudes and several others such as thrusting a hand forward with the palms shaking show a negative attitude (Lim, 2011). Besides, engagement primarily involves the movement of hands to open or close the space for the negotiation for other voices (Lim, 2011). The speed of the semiotic resources helps to express graduation. It involves fast or slow movements of the body (Hood, 2011). If graduation is fast, it shows energy, urgency and dynamism. If it is slow, it shows deliberateness and emphasis (Lim, 2011). What is meant by multimodal textual meaning is the pointing and rhythm of gesture. The former entails specificity and directionality both. The latter has to do with the interfaces that display information (Lim, 2011). It points to directional goals, probably a particular student in class or the entire class during a certain task or teaching/learning experience. Also, the pointing specificity is related to using hands, fingers, or things in class in different processes of teaching or learning.

There have been a number of studies in the SLA domain, which have used SF-MDA in different aspects of language learning. The exemplary works of research will be reviewed here. We will see that though this body of research is still limited in number, the findings have been truly valuable to the SLA theory and practice. This approach will be fit for investigating the nuances of the developmental nature of L2 affective variables, which have attracted the attention of SLA researchers more than ever before in recent years (Larsen–Freeman and Cameron, 2008; MacIntyre and Gregersen, 2012). Especially, with the advent of positive psychology in SLA studies (Dörnyei and Ryan, 2015), newer positive emotions such as enjoyment in foreign language learning have also joined the line of inquiry. Relevant studies will be exemplified here.

### Exemplary systemic functional-multimodal discourse analysis studies in second language acquisition

Peng et al. (2017) used the SF-MDA to investigate the multimodal resources of willingness to communicate (WTC) in English as a foreign language. These researchers first drew attention to the multimodal nature of human communication marked by the processing of different semiotic qualities. Using the SF-MDA, Peng et al. (2017) examined the dynamics of WTC in EFL classes in China. The data collection modes were video-recording of classes as well as student journal writing and stimulated recall interviews. The semiotic assets explored included gesture, language, and gaze. The discourse semantic features were found, analyzed and contrasted to discuss the nuanced differences. The present findings emphasize the essentiality of language teachers’ recognition and coordination of multimodal semiotic resources to improve Students’ classroom participation and WTC.

Similarly, Erfanian Mohammadi et al. (2019) examined how Teaching English as a Foreign Language students (TEFL) benefited from material and non-material semiotic assets together while engaged in the process of constructing tasks and activities for the class. They also analyzed the semiotic assets of these students such as their gestures, language, looks, using the SF-MDA. To this aim, they used an ethnography and video recorded the interaction patterns of six TEFL students who were designing materials to improve the intercultural competence of language learners about a certain general topic. The research findings showed that the participants interacted with each other as they were developing the materials. In the analysis, human semiotic resources were included as well as digital devices and also non-human assets. Besides, when they were engaged in sharing their mental processes, they also communicated positive attitudes and created some negotiation expanding space using gestures with both slow and fast graduation. The way they stared was also engaged, directed at the members of the group and also at the objects while they were designing tasks and activities for class.

Furthermore, Bayat et al. (2020) explored the enjoyment construction potential of a teacher’s multimodal negative feedback based on SF-MDA. These researchers videotaped the English language teacher’s provision of corrective feedback through multimodality with the help of non-verbal and verbal semiotic assets including posture, gaze, and gesture and monitoring the emotional experiences of language learners for several sessions. They also held interviews in the form of stimulated recalls with several learners and gathered their written journals about how they experienced enjoyment when they received the multimodal negative feedback scenarios from the teacher. SF-MDA was used to analyze the teacher’s multimodal corrective feedback. Interview and journal contents were also analyzed. The results showed that the teacher’s use of multimodality in the negative feedback added to the depth of enjoyment by drawing the Students’ attention to errors, raising their awareness of the correct forms, and raising the significance of the negative feedback.

In addition, Purwaningtyas (2020) examined the effective use of visual input in EFL textbooks using the SF-MDA. The framework employed in this study was derived from Kress van Leeuwen. The focus was on the visual representation of an EFL Indonesian book. The analysis revealed that presenting female characters visually was 70% more than the male. As for social roles, the proportion of jobs portrayed in the textbook held by men and women was to the same degree. This research aimed to examine the meaning of the incorporated employment of semiotic assets in the textbook, including the representation of visual images. This research recommended to the textbook user that the appearance of visual images can enhance the text or written content in the textbook. The findings also suggested that textbook publishers be more sensitive about the written material as well as the visual symbols used not to cause a kind of misunderstanding among the users of the textbook.

Moreover, Lim (2021) employed the SF-MDA approach to investigating the association of gesture and language the teacher used, in interacting with students, and the emergent meanings created multimodally. This study discussed the mechanisms that combined gesture and language for meaning-making by developing the concepts that were created originally for the associations of language and image. Analyzing and interpreting the teachers’ multimodal choices in the lesson led to suggesting the emergent concept of structured informality, which provides a way to facilitate how teachers can develop an efficient experience of learning for their students through multimodal assets.

### Promises of systemic functional-multimodal discourse analysis for investigating L2 affective factors

Mode refers to any semiotic resource which conveys a specific meaning. When it comes to the emotions emerging out of L2 classroom interactions, these semiotic resources can take different forms, both verbal and non-verbal, reflecting a specific emotion within the context of interaction. The existing literature on SF-MDA employed in the SLA domain shows a wide gap in the investigation of L2 affective variables. There are for sure, many more affective variables involved in language studies than WTC and foreign language enjoyment. It should be noted that these affective variables are no longer seen as traits but should be viewed from a dynamic stative perspective. This means that emotions in classroom interactions cannot be necessarily predicted based on certain conditions but the situational contexts of these interactions determine what and how emotions emerge in these contexts. Also, it is worth noting that the multimodal perspective to L2 classroom interactions should not be necessarily generalized to all contexts. Holding a culture-bound view of this multimodality can provide deeper insights into how emotions are dependent on the culture of the interlocutors involved in L2 classroom interactions. There are certain reasons why SF-MDA can and need to be used more in the inquiry of affective variables in an L2 learning context, as explained below. It should be noted that the interactions in L2 classrooms are dynamic and context-dependent. An EFL or ESL classroom is marked by the interactive development of many psychological and emotional factors. The multimodal perspective to emotions in L2 classroom interactions is not limited to any specific emotions and can be applied to all emotions in this domain. Every session, the teacher and students and the students with peers engage in different interactive tasks and activities, which can vary from one-on-one exchanges to more extensive interactions, for example, between the teacher and the whole class. These interactions can be the origin of different emotions that show emergent patterns and a fluctuating nature. The existing research in SLA, largely influenced by the complexity and dynamic systems theory (CDST) has drawn attention to the significance of tracing the developmental nature of different affective factors (e.g., enjoyment, boredom, anxiety, etc.) which can be involved in the L2 learning experience. A dynamic approach to these affective variables requires more ethnographic and longitudinal studies to be capable of tracing the complex dynamic interactions of these constructs.

As described by Dewaele and Li (2020), the SLA domain has entered its third phase of inquiry (i.e., the dynamic phase), following the general and domain-specific phases. This new phase is expected to benefit from innovative research methods which are compatible with this dynamic shift and which are capable of revealing the causes of changes in the fluctuating development of the emotional traits. The third phase of SLA research, known as the dynamic turn, shows concerns for both positive affective variables (e.g., motivation, self-confidence, grit, foreign language enjoyment) and negative constructs (e.g., demotivation, anxiety, boredom). That is, the multimodal approach to emotions in SLA can be also viewed from an inter-individual perspective. This means that each individual language learner expresses his or her own specific multimodal functions of emotions in classroom interaction. This new turn in the SLA research needs new research designs to be compatible with the CDST to approach language learners’ affective variables. Another factor which should be considered in the application of SF-MDA is the distance and status of the interlocutors in classroom interactions. That is, the multimodal functions of L2 emotions can be constructed differently whether they interact with their teachers or their classmates. A number of qualitative and quantitative research methods have been suggested for investigating different cognitive, affective and behavioral variables involved in language learning (Hiver and Al-Hoorie, 2019). Among the innovative CDST-consistent research methodologies, SF-MDA is one with a truly functional approach to classroom discourse marked by multimodality. As SF-MDA often requires multiple methods of collecting data (e.g., interview, ethnography of communication, journal writing, etc.), this approach to research is capable of showing nuances of variation in the trajectory of changes within different variables or emotions experienced by L2 teachers and learners in the natural experience of language learning embedded within the dynamic network of interactive forces in an L2 class. With the shift from a mono-modal perspective to emotions in SLA to a multimodal one, video-recording tools of classroom observations should be incorporated into research on these emotions.

## Conclusion

As already noted at the beginning of this paper, the association of discourse analysis and multimodality goes back to Kress and van Leeuwen’s multimodal discourse (2001). SF-MDA sees the concerns of discourse analysis to be inherently multimodal and reckons that this multimodality should be addressed in any study of discourse. The systemic functional theory, which is the theoretical foundation of the SF-MDA, is marked by amenability and adaptability (Martin, 2002). Yet, it should be reminded that though the higher-level principles of the systemic functional theory can be used for analyzing multimodal texts, the systems for visual input and other semiotic assets are not the same as those for language. The SF-MDA goes beyond the mere extension of the well-known SF-approaches that have been mainly proposed to model discourse and grammatical systems of language (O’Halloran, 2008), and entails the generation, and incorporation of different but complementary approaches and models for investigating multimodal meaning-making.

When used in the SLA domain, SF-MDA can be hoped to represent a more realistic, all-inclusive analysis of multiple modes of interaction that language learners involve in. The kind of communication that language learners have in class helps them practice the language they are learning and also develop interpersonal communication skills. The CDST reckons that not only the learning occurs through this complex and dynamic network of interactions, but also the affective constructs emerge out of this complexity. Thus, analyzing the teacher-student and student-student interactions in L2 classes through the SF-MDA approach can show how language learners experience different intensities of certain affects in different moments of the multimodal interactions. SF-MDA allows researchers to learn more about L2 learners’ experience of affective variables in the immediate learning context through assessing the individual Students’ perceptions, emotions, evaluations, and thoughts as they are engaged in the one-on-one, one-on-whole class or group-based communicative tasks and activities. SF-MDA manages to capture affect, experience, and behavior in the real time through several methods of data collection. Thus, it gives SLA researchers a way to extend their knowledge of language learners’ perceived emotions they can explore and describe and to better make sense of how students and the different aspects of the learning environment hand in hand form such experiences.

This paper showed why SF-MDA could be of particular value to educational investigations and the SLA line of research by allowing researchers to formulate and answer new research questions about how language learners, their peers, and their teacher, all involved in the language learning context, develop certain emotions. SF-MDA, thus, helps us to better make sense of how several modes of input and interaction influence learning and the other relevant outcomes. The present paper pinpointed the value of the innovative SF-MDA approach in seeking answers to new and interesting questions which can assist SLA researchers and practitioners to explore nuances of the systematic and dynamic variation of affective variables as they actually emerge out of the multimodal interactions in an L2 class.

It is noteworthy that the SF-MDA and other similar innovative approaches to research are well adapted to the CDST requirements of inquiry. Thus, they are expected to be used more in the present era, heavily influenced by the complexity and dynamicity of human cognition, emotion and behavior emerging out of the multimodal interactions. SLA domain should also keep up with the ever-growing movement, and is expected to benefit from the power of the SF-MDA approach in tracing the dynamicity of the L2 affective variables, which are mostly under-researched in the dynamic phase of the SLA research. Finally, it should be reminded that the distinctive feature of the SF-MDA is multimodality at its core, which is becoming more and more interesting to researchers in the applied linguistics domain. In its underlying theory, the SF-MDA has the significant role of actors within a network (compatible with the CDST). This approach can be used to explore how L2 learners take advantage of textual and non-textual semiotic assets to develop and express their emotions when they have engaged actively in classroom tasks and activities. Furthermore, the semiotic approach places emphasis on the functional role of photos which can serve as vehicles of cultural knowledge integrated with texts and pedagogic tasks. They are more than decorative tools (Derakhshan, 2021). Consequently, the semiotic assets of the L2 learners such as their language, the way they look, their facial expressions and body gestures can be analyzed in depth using the SF-MDA approach, and can significantly contribute to the existing literature.

Regarding the pedagogical implications of the outcomes of the SFMA studies, language teachers can foster embodied or multimodal classroom interactions in the setting of their classes with a focus on how emotions emerge out of these interactions. Moreover, given the stative and situational nature of L2-related emotions, they should be sensitive to how and what emotions are reflected in the momentary student-student and teacher-student interactions. Furthermore, L2 learners’ awareness should be raised on how they can make congruence between their gesture and their emotions and how they can interpret their interlocutors’ emotions in dyadic classroom interactions by being sensitive to their non-verbal feedback. With respect to the limitations of this review, it should be mentioned that this review has been mainly developed from a methodological perspective but the arguments for the application of SF-MDA in L2 affective variables can be also developed from different perspectives.

## Suggestions for further research

Paying attention to the multimodal aspect of emotions in the L2 context should be seen as an inseparable aspect of researching these emotions. This means that exploring different aspects of L2 emotions in future studies should be carried out with a multimodal lens. The main implication of this study for those who engaged in the language teaching or learning experience is the recognition of the multiple modes of communication in an L2 class, which can be explored or analyzed hand in hand. Indeed, the emotions that L2 learners develop emerge from these multimodal language learning episodes. The affective variables that develop from this multimodal learning experience are not stable. Rather, they are changing meaningfully in response to different modes of feedback received from the teacher, peer, or other elements of the environment of learning. It is worth investigating these patterns of emergent emotions as they actually occur in class by recording them for future analysis. These changing patterns in the emotions in the live experience of classroom learning can have different causal mechanisms, which can be explored using SF-MDA as well. For example, getting to know why certain modes of feedback (provided by the teacher) fail to motivate low-achievers in class is worth investigating. Or, for another instance, seeing what modes of corrective feedback can work best to prevent social aloofness or boredom is worth investigating too. The findings from the SF-MDA approach to L2 affective variables can be used to make EFL/ESL teachers better aware of the multimodality of communication in L2 classrooms, the effectiveness of considering this multimodality in motivating students to learn L2 better, to better adapt the session content and the tasks and activities included to preclude negative emotions and contribute to the positive ones.

EFL/ESL teachers can be better prepared to see different patterns of, for instance, enjoyment or boredom in class from different students, and not to use the same modes of interaction for (or do not expect to see the same modes of interaction from) all students. Until now, many language learning emotions are still unexplored through the SF-MDA approach (e.g., anxiety, passion for learning, boredom), which need to be investigated systematically, functionally and dynamically, to further enrich the dynamic phase of the SLA line of research. The findings will hold promises for a more realistic conceptualization of learning with all human and emotional aspects of learning realized through multimodal communication in an L2 class.

MDA has been categorized as a branch of discourse analysis, which examines language together with other modes of communication (e.g., pictures, body language, sounds, and icons). Consequently, one can use SF-MDA as an effective analysis framework of analysis since it involves several functions and systems used to create meaning through various resources. Therefore, it can be concluded that the SF-MDA is capable of investigating the multimodality of communication, which is the main purpose of language use. It can reveal how affective variables also develop naturally out of these multimodal language-mediated interactions in class. It closely monitors language learners’ language, gestures, looks and interactions within the interactive tasks and activities embedded in classroom learning. It often uses multiple ways of data collection such as interviews, videorecording, stimulated recalls, journal writing and the like to ensure the inclusion of all details possible and not to miss any important point in the analysis. The authentic moment-by-moment tracing of how language learners develop a certain emotion (e.g., boredom, enjoyment, etc.) while involved in communicative tasks and activities in an L2 class can have different implications for the SLA domain too.

A brief look at the related literature on SF-MDA in SLA research shows that the existing body of research on L2 affective variables is still in its infancy. Moreover, the few studies conducted so far using the SF-MDA approach in examining some L2 affective variables have only emerged within the past 5 or 6 years. Therefore, we can conclude that the use of SF-MDA has been only recently taken seriously in the SLA domain, and still has a long way ahead in research. Since SF-MDA has been recently introduced to the field of SLA, some researchers interested in the investigation of L2 emotions might be still unfamiliar with the procedures of this methodological approach. Thus, they need to improve their literacy in the use of this method. However, the results of the limited SF-MDA studies conducted to explore L2 affective variables have made significant contributions to the CDST-guided line of research in SLA domain, especially in the dynamic phase. SF-MDA, with its distinctive advantages and the use of ethnographies, is capable of delineating the complex growth and the fluctuations of language learners’ emotions during the actual experience of language learning. Therefore, we can hope to see more SF-MDA studies in near future to explore different aspects of language learners’ emotional experiences emerging from and within communications. Why SF-MDA is matched with the dynamic studies of L2 affective variables and what promises it holds for the domain were already addressed. A number of research questions were also suggested which can all be used to direct the future line of SF-MDA studies. Here are several exemplary research questions that can possibly be addressed in SLA research to explore L2 affective variables from the SF-MDA approach:

- What is the L2 learners’ multimodal expression of (affective variable e.g., boredom, enjoyment, anxiety) during a certain task or activity in class?
- What is the teacher’s multimodal feedback to reduce or remove a negative L2 affective variable (e.g., anxiety, boredom, stress) expressed by students in a certain task or activity?
- What are the multimodal manifestations of a certain positive affective variable (e.g., enjoyment) in student-student pair or group works in class? How can the teacher feed into this expression of positive emotions?
- What are the multimodal manifestations of a certain negative affective variable (e.g., boredom) in student-student pair or group works in class? How can the teacher reduce this expressed negative emotions without being obtrusive?
- Which aspect(s) of the multimodality of expressing L2 affective variables are more culture-bound in a certain context? And why?
- Is there any cross-gender difference in the use of physical gesture in expressing a certain emotion in an L2 class in (a particular country)? Does it have any cultural root(s)?
- What compensatory strategies can be used to increase the multimodal aspects of interactions in L2 classes especially in EFL settings with a far different cultural roots from the L2 culture and context?
- What is the attitude of preservice L2 teachers toward the relationship between the multimodality of L2 class resources (tasks, activities, materials) and the varying degrees of emotions they raise in language learners?
- How can the multimodality of the learning materials influence the further intensification of language learners’ positive emotions (e.g., motivation, high self-confidence, foreign language enjoyment)?
- How can the multimodality of the learning materials influence the lowering of language learners’ negative emotions (e.g., boredom, anxiety, stress)?
- How can multimodality be used in the design of language tests so as to minimize the affective filters and maximize the positive emotions?
- How can learner-developed multimodal materials, tasks or activities be incorporated in class events so that the affective filter is lowered and the positive emotions are intensified instead?
- How can multimodal classroom learning input be incorporated so as to motivate cultural minorities in class (among students) and to increase their willingness to communicate?
- How can the teacher possibly model the best advantages taken of the multimodal semiotics in an L2 class so that students feel safe to follow?
- What inhibitions and limitations can possibly be set to the extended use of multimodal semiotics in L2 classes in developing countries (possibly by the curriculum designers or educational policy makers)?

Exploring these research questions can even require mixed designs. Thus, quantitative and qualitative lines of inquiry are both needed to explore the functionalities of language use in real communication, and also, as we contend, for the communication rehearsed in l2 classrooms. As the emergence and development of affective variables are marked by dynamicity, either the quantitative or qualitative approach (or even the mixed approach) taken should be compatible with the CDST-guided line of research. Also, SFMA can contribute to the understanding of unexplored emotional phenomena such as emotion contagion like enjoyment contagion (see Talebzadeh et al., 2020).

## Author contributions

The author confirms being the sole contributor of this work and has approved it for publication.

## References

[B1] Alyousef H. S. (2020). A multimodal discourse analysis of English dentistry texts written by Saudi undergraduate students: A study of theme and information structure. *Open Linguist.* 6 267–283. 10.1515/opli-2020-0103

[B2] Baldry A. P. Thibault P. J. (2006). *Multimodal Transcription and Text.* London: Equinox.

[B3] Bateman J. (2008). *Multimodality and Genre: A Foundation for the Systematic Analysis of Multimodal Documents.* Basingstoke: Palgrave Macmillan. 10.1057/9780230582323

[B4] Bayat M. Elahi Shirvan M. Barabadi E. (2020). A teacher’s multimodal corrective feedback: Exploring its enjoyment building capacity. *Pol. Psychol. Bull.* 51 71–88.

[B5] Chen Y. (2010). The semiotic construal of attitudinal curriculum goals: Evidence from EFL textbooks in China. *Linguist. Educ.* 21 60–74. 10.1016/j.linged.2009.12.005

[B6] Derakhshan A. (2021). Should textbook images be merely decorative?’: Cultural representations in the Iranian EFL national textbook from the semiotic approach perspective. *Lang. Teach. Res.* 25 1–35. 10.1177/1362168821992264

[B7] Dewaele J. M. Li C. (2020). Emotions in second language acquisition: A critical review and research agenda. *Foreign Lang. World* 196 34–49.

[B8] Dörnyei Z. Ryan S. (2015). *The Psychology of the Language Learner Revisited.* London: Routledge. 10.4324/9781315779553

[B9] Erfanian Mohammadi J. Elahi Shirvan M. Akbari O. (2019). Systemic functional multimodal discourse analysis of teaching students developing classroom materials. *Teach. High. Educ.* 24 1–23. 10.1080/13562517.2018.1527763

[B10] Halliday M. A. K. (1978). *Language as Social Semiotic: The Social Interpretation of Language and Meaning.* London: Edward Arnold.

[B50] Halliday M. A. K. (1985). *An Introduction to Functional Grammar.* London: Edward Arnold.

[B11] Halliday M. A. K. (2004). “Representing the child as a semiotic being,” in *Language, Education and Discourse: Functional Approaches*, ed. Foley J. (London: A&C Black), 19–42.

[B12] Halliday M. A. K. Matthiessen C. M. I. M. (1999). *Construing Experience Through Meaning: A Language Based Approach to Cognition.* London: Cassel.

[B13] Hiver P. Al-Hoorie A. H. (2019). *Research Methods for Complexity Theory in Applied Linguistics.* Bristol, UK: Multilingual Matters. 10.21832/HIVER5747

[B14] Hood S. (2011). “Body languages in face-to face teaching: A focus on textual and interpersonal meaning,” in *Semiotic Margins: Meaning in Modalities*, eds Dreyfus S. Hood S. Stenglin M. (London: Continuum), 31–52.

[B15] Iedema R. (2001). Resemiotition. *Semiotic* 137 23–39. 10.1515/semi.2001.106

[B16] Iedema R. (2003). Multimodality. resemioticization: Extending the analysis of discourse as a multiperiodic practice. *Vis. Commun.* 2 29–57. 10.1177/1470357203002001751

[B17] Jewitt C. (2011). *The Routledge Handbook of Multi Modal Analysis.* London: Routledge.

[B18] Kiss T. Weninger C. (2017). Cultural learning in the EFL classroom: The role of visuals. *ELT J.* 71 186–196. 10.1093/elt/ccw072

[B19] Kramsch C. (2013). Culture in foreign language teaching. *Iran. J. Lang. Teach. Res.* 1 57–78.

[B20] Kress G. Van Leeuwen T. (2001). *Multimodal Discourse: The Modes and Media of Contemporary Communication Discourse.* London: Arnold.

[B21] Kress G. van Leeuwen T. (2006). *Reading Images: A Grammar of Visual Design.* London: Routledge. 10.4324/9780203619728

[B22] Larsen–Freeman D. Cameron L. (2008). *Complex Systems and Applied Linguistics.* Oxford: Oxford University Press.

[B23] Lemke J. L. (1998). “Multiplying meaning: Visual and verbal semiotics in scientific text,” in *Reading Science: Critical and Functional Perspectives on Discourses of Science*, eds Martin J. R. Veel R. (London: Routledge), 87–113.

[B24] Levasseur D. G. Sawyer J. K. (2006). Pedagogy meets PowerPoint: A research review of the effects of computer-generated slides in the classroom. *Rev. Commun.* 6 101–123. 10.1080/15358590600763383

[B25] Lim F. V. (2011). *A Systemic Functional Multi Modal Discourse Analysis Approach to Pedagogic Discourse*, Ph.D. thesis, Singapore: National University of Singapore.

[B26] Lim F. V. (2021). Investigating intersemiosis: A systemic functional multimodal discourse analysis of the relationship between language and gesture in classroom discourse. *Vis. Commun.* 20 34–58. 10.1177/1470357218820695

[B27] MacIntyre P. D. Gregersen T. (2012). Emotions that facilitate language learning: The positive–broadening power of the imagination. *Stud. Second Lang. Learn. Teach.* 2 193–213. 10.14746/ssllt.2012.2.2.4

[B28] Martin J. R. (1992). *English Text: System and Structure.* Amsterdam: John Benjamins. 10.1075/z.59

[B29] Martin J. R. (2002). “Fair trade: Negotiating meaning in multimodal texts,” in *The Semiotics of Writing: Transdisciplinary Perspectives on the Technology of Writing*, ed. Coppock P. (Turnhout: Brepols), 311–338.

[B30] Martin J. R. Rose D. (2003). *Working With Discourse: Meaning Beyond the Clause.* London: Continuum.

[B31] Martin J. R. White P. R. R. (2005). *The Language of Evaluation: Appraisal in English.* London: Palgrave Macmillan. 10.1057/9780230511910

[B32] Martinec R. (2005). A system for image–text relations in new media. *Vis. Commun.* 4 337–371. 10.1177/1470357205055928

[B33] O’Halloran K. L. (2004). *Multimodal Discourse Analysis.* London: Continuum.

[B34] O’Halloran K. L. (2005). *Mathematical Discourse: Language, Symbolism and Visual Images.* London: Continuum.

[B35] O’Halloran K. L. (2007). “Systemic functional multimodal discourse analysis (SF-MDA) approach to mathematics, grammar and literacy,” in *Advances in Language and Education*, eds McCabe A. O’Donnell M. Whittaker R. (London: Continuum), 75–100.

[B36] O’Halloran K. L. (2008). Systemic functional-multimodal discourse analysis: Constructing ideational meaning using language and visual imagery. *Vis. Commun.* 7 443–475. 10.1177/1470357208096210

[B37] O’Halloran K. L. (2011). “Multimodal discourse analysis,” in *Companion to Discourse*, eds Hyland K. Paltridge B. (London: Continuum), 120–137.

[B38] O’Toole M. (1994). *The Language of Displayed Art.* London: Leicester University Press.

[B39] Peng J. E. Zhang L. Chen Y. (2017). The mediation of multimodal affordances on willingness to communicate in the English as a foreign language classroom. *TESOL Q.* 51 302–331. 10.1002/tesq.298

[B40] Purwaningtyas T. (2020). Didactic symbol of visual images in EFL textbook: Multi-modal critical discourse analysis. *Pedagogy* 8 51–63. 10.32332/pedagogy.v8i1.1959

[B41] Rowsell J. Collier D. R. (2017). “Researching multimodality in language and education,” in *Research Methods in Language and Education*, eds King K. A. Lai Y. J. May S. (Berlin: Springer International Publishing), 311–324. 10.1007/978-3-319-02249-9_23

[B42] Royce T. (1998). Synergy on the page: Exploring intersmiotic complementarity in page-based multimodal text’. *JASFL Occas. Pap.* 1 25–49.

[B43] Talebzadeh N. Elahi Shirvan M. Khajavy G. H. (2020). Dynamics and mechanisms of foreign language enjoyment contagion. *Innov. Lang. Learn. Teach.* 14 399–420. 10.1080/17501229.2019.1614184

[B44] Unsworth L. (2001). *Teaching Multiliteracies Across the Curriculum: Changing Contexts of Text and Image in Classroom Practice.* Buckingham: Open University Press.

[B45] Unsworth L. (2006). Towards a metalanguage for multiliteracies education: Describing the meaning making resources of language-image interaction. *English Teach.* 5 55–76.

[B46] Van Leeuwen T. (1999). *Speech, Music, Sound.* London: Macmillan. 10.1007/978-1-349-27700-1

[B47] Van Leeuwen T. (2005). *Introducing Social Semiotics.* London: Routledge. 10.4324/9780203647028

[B48] Ventola E. Charles C. Kaltenbacher M. (2004). *Perspectives on Multimodality.* Amsterdam: John Benjamins. 10.1075/ddcs.6

[B49] Wang Y. Hemchua S. (2022). Can we learn about culture by EFL textbook images?: A semiotic approach perspective. *Lang. Relat. Res.* 13 479–499. 10.52547/LRR.13.3.19

